# Impact of oxidative stress on malignant tumor progression and emerging therapeutic strategies

**DOI:** 10.3389/fmolb.2026.1916698

**Published:** 2026-08-21

**Authors:** Xudong Feng, Guicai Liang, Jingjing Sun, Zhiqiang Wang, Tao Li, Yang Fu, Wen Deng, Xiuying Li, Jie Luo, Ping Jin, Chunlei Ge, Rongqing Li

**Affiliations:** 1 Department of Radiation Oncology, The First Affiliated Hospital of Kunming Medical University, Kunming, China; 2 Cancer Biotherapy Center, The Third Affiliated Hospital of Kunming Medical University, Yunnan Cancer Hospital, Peking University Cancer Hospital Yunnan, Kunming, China; 3 Department of Child Healthcare, Kunming Children’s Hospital, Kunming, Yunnan, China; 4 State Key Laboratory for Conservation and Utilization of Bio-Resources in Yunnan, School of Life Sciences, Yunnan University, Kunming, Yunnan, China

**Keywords:** malignant tumors, oxidative stress, progression, reactive oxygen species, therapy

## Abstract

Oxidative stress, driven by an imbalance between reactive oxygen species (ROS) production and antioxidant defenses, plays a pivotal role in cancer biology. While persistent, moderately elevated ROS levels can promote genomic instability, tumor progression, and immune evasion, excessive ROS accumulation can overwhelm antioxidant defenses and trigger cancer cell death. Despite promising preclinical findings, clinical translation remains a challenge because of the context-dependent effects of ROS. A deeper understanding of oxidative stress regulation may lead to the development of novel precision medicine strategies, optimizing cancer therapies while minimizing adverse effects. This review highlights the concentration-dependent effects of oxidative stress in tumorigenesis, focusing on its impact on DNA damage, metabolic reprogramming, and immune modulation. We discuss recent advancements in ROS-targeting strategies, including pro-oxidant therapies that exploit redox vulnerabilities in cancer cells and antioxidant-based approaches aimed at mitigating oxidative stress-driven resistance to treatment. Moreover, we examine the interplay between oxidative stress and the tumor microen-vironment, emphasizing its influence on immune surveillance and therapeutic responses. This re-view provides insights into ROS-targeting interventions and their potential in oncologic treatment paradigms.

## 1 Introduction

Cancer is a disease caused by uncontrolled and disordered cell proliferation, which may spread to other parts of the body (Din et al., 2023). Unlike normal cells, which follow strict growth and division rules, cancer cells lose these control mechanisms, resulting in tumor formation (Biffi and Tuveson, 2021; Marti and Tuveson, 2021). The genesis of cancer is a multifaceted process that is not only related to genetic background but also influenced by various factors, such as environment and personal living habits (Leiter et al., 2023; Cai et al., 2021; Guo et al., 2021; Machlowska et al., 2020). Among these factors, oxidative stress plays a complex and critical role (Wu et al., 2022; Lin et al., 2019). Oxidative stress is defined as a disturbance in the equilibrium between reactive oxygen species (ROS) generation and the body’s antioxidant defense mechanisms, resulting in ROS accumulation that surpasses the body’s ability to remove them. Elevated ROS levels can be cytotoxic; compared with normal cells, cancer cells generally exhibit high ROS status. This imbalance can detrimentally affect cellular structure and function (Teleanu et al., 2022; Forman and Zhang, 2021). In particular, it can jeopardize genomic stability and interfere with cellular signaling pathways, processes that are closely associated with the initiation and progression of cancer (Perillo et al., 2020).

Current cancer treatment modalities primarily include surgery, chemotherapy, targeted therapy, and immunotherapy (Kaur et al., 2023; Passaro et al., 2024). During these interventions, cancer cells are often subjected to persistent oxidative stress by increasing the levels of ROS. Within the realm of oncology, modulating oxi-dative stress is deemed a pivotal therapeutic strategy. Consequently, targeting antioxidant mech-anisms within tumor cells might yield beneficial therapeutic outcomes (Li K. et al., 2024; Li et al., 2023; Moloney and Cotter, 2018; Sun et al., 2023; Wang et al., 2021). Moreover, a complex interplay exists between oxidative stress and tumor immunity. In immune cells, moderate ROS escalation enhances metabolism and facilitates the synthesis and secretion of cytokines and other effector molecules. Conversely, in cancer cells, elevated ROS levels and immunosuppressive agents, such as programmed death-ligand 1 (PD-L1) and lactate, can suppress immune cell function. This intricate balance underscores the potential of targeting oxidative stress pathways as a therapeutic avenue in cancer treatment (Cribbs et al., 2020; Kroemer et al., 2022; Mantovani et al., 2019; Peiseler et al., 2022; Sies and Jones, 2020).

Through a comprehensive examination of the concentration-dependent effects of ROS in cancer progression and therapy, we can leverage this knowledge to guide the development of innovative therapeutic strategies. In this review, we explore the impact of ROS on the initiation and progression of malignant tumors, alongside recent developments in cancer treatment methodologies.

Over the past several years, the field of ROS in cancer has witnessed an explosion of high-quality reviews covering various aspects of this complex topic. Foundational overviews have systematically summarized the basic mechanisms of ROS generation and their cytotoxic effects in cancer therapy (Sahoo et al., 2022). Comprehensive reviews have also addressed the design and application of ROS-scavenging nanomaterials in diverse diseases, including cancer (Zhang et al., 2022; Zhang et al., 2025). Most recently, Shen et al. provided a thorough analysis of ROS in the tumor microenvironment, covering cell death, metabolic reprogramming, and emerging therapeutic modalities such as NDDS, PROTAC, and CAR-T therapy (Shen et al., 2025), while Akter et al. delivered an exceptionally comprehensive synthesis of ROS biology, signaling pathways, metabolism, cell death, and therapeutic strategies in cancer (Akter et al., 2026). In parallel, Li et al. proposed the elegant “ROS-Immune Mismatch” framework, articulating the differential redox sensitivities between tumor cells and immune subsets that drive immunosuppression (Li et al., 2026), and Sahu et al. systematically classified ROS-modulating compounds according to their predominant signaling pathways (Sahu and Jain, 2026). Other specialized reviews have focused on ROS in metastasis (Gilchrist et al., 2026), the Nrf2-ROS-EMT network (Xu et al., 2025), ROS-mediated cell death in specific cancer types (Wei et al., 2025), and the bidirectional crosstalk between ROS and DNA epigenetic modifications (Yang et al., 2026; Tao et al., 2024).

Building upon these outstanding contributions, our review distinguishes itself through several unique features. First, we adopt the three-tier concentration-dependent model (basal, moderate, and excessive ROS) as an overarching organizing framework to explain the paradoxical roles of ROS across different biological contexts from tumor initiation and progression to immune modulation and therapeutic response. Second, we integrate mechanistic advances spanning ROS-mediated epigenetic regulation (including DNA methylation and histone modifications via DNMTs and TETs (Yang et al., 2026)), organelle-specific redox signaling, and the crosstalk with regulated cell death pathways (ferroptosis, pyroptosis, and necroptosis). Third, we provide a systematic classification of ROS-targeting therapeutic agents, from small-molecule pro-oxidants and antioxidant inhibitors to nanomedicines and PROTACs, with a summary of their clinical trial status, complementing the pathway-centric classification of Sahu and Jain (2026) with a more clinically oriented perspective. Fourth, and most importantly, we critically discuss the translational challenges including patient stratification, off-target toxicity, NRF2-driven resistance, the antioxidant paradox, and the spatiotemporal complexity of ROS regulation that have limited the clinical progress of ROS-based therapies. These insights may hold significant implications for clinical practice, offering tangible benefits in cancer treatment.

## 2 Oxidative stress and reactive oxygen species

Oxidative stress refers to the disruption of the balance between the production and removal of free radicals, particularly ROS, such as superoxide anions and hydrogen peroxide, within living organisms. This imbalance leads to excessive accumulation of free radicals, causing damage to cellular structures and impairing physiological functions (Forman and Zhang, 2021; Bai et al., 2022). ROS can trigger reversible oxi-dative modifications on critical cysteine residues within target proteins (Lennicke and Cochemé, 2021), posttranslational modifications (PTMs) that play pivotal roles in regulating the biological activities of various en-zymes and transcription factors. PTM not only influences the intracellular localization of these enzymes and transcription factors but also modulates their interactions with partner molecules (Sies and Jones, 2020; Xu Y. et al., 2024). Moreover, ROS are closely connected to cellular metabolic activities, influencing energy metabolism and the function of metabolic enzymes, thereby impacting cell growth and differenti-ation. In addition, ROS are vital in regulating gene expression, as they affect the activity and stability of transcription factors, which in turn influence cellular functionality and survival (Sies and Jones, 2020; Chen et al., 2021). Further, ROS can react with proteins and DNA, causing protein oxidation and DNA damage, which can impair normal cellular function and even trigger cell death (Liu T. et al., 2022; Kajarabille and Latunde-Dada, 2019).

The generation of ROS is categorized into two types: endogenous and exogenous. Endogenous ROS originate primarily from cellular oxidative metabolic processes, including oxidative phos-phorylation and electron transfer chain reactions (Wang et al., 2021; Yang et al., 2019; Castelli et al., 2021; Jomova et al., 2024; Leuti et al., 2019), which mainly occur in various met-abolic activities of cellular organelles, such as mitochondria, peroxisomes, and the endoplasmic reticulum (Juan, 2021; Moldogazieva et al., 2020). Approximately 2% of the oxygen consumed by the mitochondria is reduced to form superoxide, making mitochondria a major source of ROS (Cadenas, 2018; Palma et al., 2024a; Zhou and Tian, 2018). Under physiological conditions, ROS play a critical role in various cellular functions and are considered signaling molecules or second messengers in cellular signaling pathways, a process referred to as redox signaling (Lennicke and Cochemé, 2021; Angelova and Abramov, 2018; Jomova et al., 2023; Noctor et al., 2018; Peltor et al., 2018).

Organelle-specific ROS signaling is not uniform but is determined by the site of ROS generation and local redox-buffering capacity. In mitochondria, electron leakage from respiratory complexes I and III generates superoxide and H_2_O_2_. Under mild hypoxia, mitochondrial ROS inhibit prolyl hydroxylase domain proteins and stabilize HIF-1α, thereby promoting glycolytic adaptation, angiogenesis, epithelial-mesenchymal transition, and metastatic dissemination. A recent melanoma study further identified a cyclophilin D-mitochondrial permeability transition pore-mitochondrial Ca^2+^-ROS positive feedback circuit that sustains pseudohypoxic HIF-1α activation (Bae et al., 2024; Park et al., 2025). Mitochondrial dysfunction additionally integrates respiratory chain ROS with Ca^2+^ dyshomeostasis, metabolic rewiring, and organelle quality control programs (Zong et al., 2024). In particular, the mitochondrial unfolded protein response (UPRmt) can restore proteostasis and support tumor cell survival under moderate oxidative stress, whereas unresolved or excessive stress reinforces ROS production, mitophagy, and apoptosis (Zhang X. et al., 2024). In the endoplasmic reticulum (ER), oxidative protein folding through the protein disulfide isomerase/ERO1 system generates H_2_O_2_. Accumulation of misfolded proteins activates the PERK-eIF2α-ATF4 arm of the unfolded protein response: transient signaling supports antioxidant adaptation through NRF2 and amino acid/redox metabolism, whereas persistent stress induces CHOP, ERO1α-dependent ROS amplification, mitochondrial Ca^2+^ overload, and apoptosis (Zhang et al., 2019). This ER-to-mitochondria propagation is facilitated at mitochondria-associated ER membranes, where the IP3R-GRP75-VDAC-MCU complex transfers Ca^2+^ into mitochondria and couples ER stress to mitochondrial ROS production and cell-fate decisions (An et al., 2024). Peroxisomes also generate H_2_O_2_ during fatty-acid oxidation, and altered PPAR-peroxisome signaling can convert this compartment into a therapy-resistance node. In prostate cancer, androgen-receptor inhibition activates a PPARα/PKC-dependent peroxisomal ROS program that contributes to enzalutamide resistance (Shiota et al., 2024). More broadly, recent redox-regulation frameworks show that organelle-derived ROS converge on redox-sensitive Ca^2+^, AMPK, mTOR, and NRF2 signaling nodes, thereby linking the subcellular origin of ROS to metabolic adaptation and therapeutic vulnerability (Li et al., 2025). These examples illustrate that organelle identity and interorganelle communication help determine whether ROS initiate adaptive, metastatic, therapy-resistant, or cell-death programs.

Within cells, maintaining a delicate equilibrium between the generation and removal of ROS is vital. This balance ensures the precise regulation of ROS concentrations and allows them to function effectively as secondary messengers in cellular signaling pathways (Wang and Zhang, 2024). However, when ROS are produced abnormally or when the regulatory mechanisms that inhibit excessive ROS production fail, disruption of the intracellular redox balance can occur. Excessive ROS may trigger potential toxic effects, including oxidative damage to nucleic acids (DNA and RNA), lipids, and proteins, as well as cell membrane destruction caused by lipid peroxidation. These effects may contribute to various diseases, including cancers (Lee et al., 2019; Liu J. et al., 2023; Singer et al., 2021; Vogel et al., 2020).

To protect cells from oxidative damage, various antioxidant defense mechanisms exist in the body. This mechanism involves antioxidant enzymes, such as superoxide dismutase (SOD) (Zhang et al., 2018), which converts superoxide anions into hydrogen peroxide that is subsequently decomposed into water and oxygen by catalase or glutathione peroxidase (GPX) (Sies and Jones, 2020; Okoye et al., 2023). In addition to enzymatic antioxidants, nonenzymatic antioxidants within cells, such as vitamin C, vitamin E, glutathione (GSH), and coenzyme Q10, can directly scavenge free radicals or promote the regeneration of other antioxidants (Conrad and Pratt, 2019; Zhang et al., 2023). Furthermore, oxidative stress can be modulated through multiple mechanisms, such as alterations in gene expression, activation or suppression of signaling pathways, and mod-ifications of dietary and lifestyle habits (Baird and Yamamoto, 2020; Liu et al., 2024; Zhou H. et al., 2024).

In summary, oxidative stress results from an imbalance between the production and clearance of free radicals. ROS affect protein function via oxidative modification, which regulates metabolism and gene expression. Disruption of the physiological balance of ROS can damage DNA, lipids, and proteins, leading to disease (Liu J. et al., 2023; Mittler et al., 2022; Song et al., 2020; Jelic et al., 2021; Klaunig, 2018; Kuo et al., 2022).

## 3 Oxidative stress and tumors

When the balance between the production of ROS and the cellular antioxidant defense system is disrupted, oxidative stress predisposes individuals to various diseases, including cancer, neu-rodegenerative diseases, and cardiovascular diseases. However, the role of ROS in the initiation, progression, and treatment of cancer remains controversial. To maintain abnormal proliferation, cancer cells need a large amount of adenosine triphosphate (ATP), which inevitably leads to the accumulation of ROS. Therefore, cell survival can be ensured by enhancing the clearance mecha-nisms of ROS. Studies indicate that ROS may play a dual role in carcinogenesis and tumor sup-pression under different conditions (Jelic et al., 2021; Kuo et al., 2022).

### 3.1 ROS generation and tumorigenesis

ROS contribute to cancer progression through several mechanisms. First, ROS can interact with DNA, causing several types of damage, including single-strand breaks, double-strand breaks, strand disruptions, and base alterations. Such DNA damage can result in mutations and genomic instability, which are pivotal in cancer development (Jakubczyk et al., 2020; Srinivas et al., 2019). Second, ROS can initiate signaling pathways, including the MAPK and NF-κB pathways, within cancer cells, which can consequently increase cell proliferation, survival, and migration (Moloney and Cotter, 2018; Cui et al., 2017; DeBerardinis and Chandel, 2016; Du et al., 2022). Third, ROS can compromise the function of immune cells, including T cells, natural killer cells, and macrophages, leading to an immunosuppressive state. This weakened immune environment enables cancer cells to evade immune detection, thereby facilitating their proliferation (Xie et al., 2023). Fourth, ROS can facilitate angio-genesis, the process of forming new blood vessels, thereby supplying cancer cells with essential nutrients and oxygen, and consequently, fostering tumor growth and metastasis (Cui et al., 2017; Hutchings et al., 2023). Finally, ROS may contribute to cancer resistance to treatment, such as radiation and chemotherapy, as cancer cells with elevated ROS scavenging capabilities can endure the DNA damage induced by these treatments. Recent work further showed that loss of polynucleotide kinase/phosphatase causes persistent cytosolic DNA accumulation and that ROS production synergizes with this repair deficiency to activate type I interferon signaling, illustrating a mechanistic link between oxidative DNA damage and innate immune responses (Kate et al., 2024).

**FIGURE 1:**
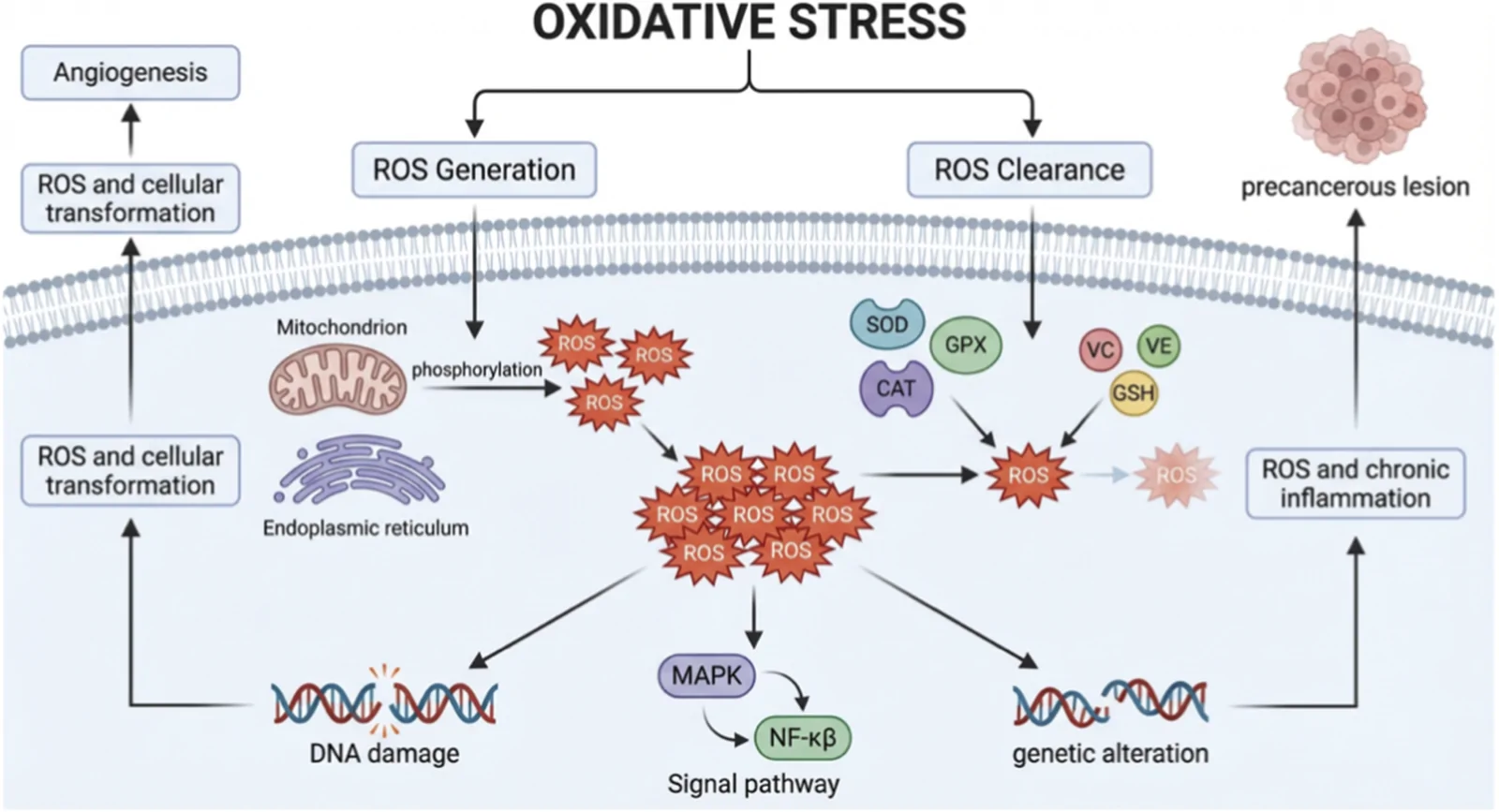
The role of oxidative stress in cellular damage during cancer development. Oxidative stress, which arises from an imbalance between the generation and clearance of reactive oxygen spe-cies (ROS), frequently contributes to tumor formation. Intracellular sources, such as mitochondria and the endoplasmic reticulum, as well as extrinsic factors like chronic inflammation, contribute to ROS production. To counteract this, cells employ a defense system composed of antioxidant enzymes (e.g., SOD, GPX, CAT) and antioxidants (e.g., Vitamin C, Vitamin E, GSH). When ROS generation over-whelms the cell’s clearance capacity, the resulting oxidative stress can lead to severe cellular damage. This includes direct DNA damage, disruption of critical signaling pathways like MAPK and NF-κB, and genetic alterations. Downstream consequences of prolonged oxidative stress include cellular transformation, angiogenesis, and the development of precancerous lesions and the progression of cancer.

In addition, the mechanism of carcinogenesis caused by ROS may be related to the formation of precancerous lesions caused by continuous chronic inflammation. In chronic inflammatory cells, the massive secretion of ROS results in the recruitment of more activated immune cells, ultimately leading to precancerous lesions. If increased intracellular ROS levels are sufficient to overcome the endogenous antioxidant response, irreversible oxidative damage to nucleic acids, lipids, and proteins may trigger genetic and/or epigenetic changes, leading to the dysregulation of oncogenes and tumor suppressor genes (Figure 1) (Larabi et al., 2020; Racanelli et al., 2018).

### 3.2 Dysregulation of the antioxidant defense system and tumorigenesis

**FIGURE 2:**
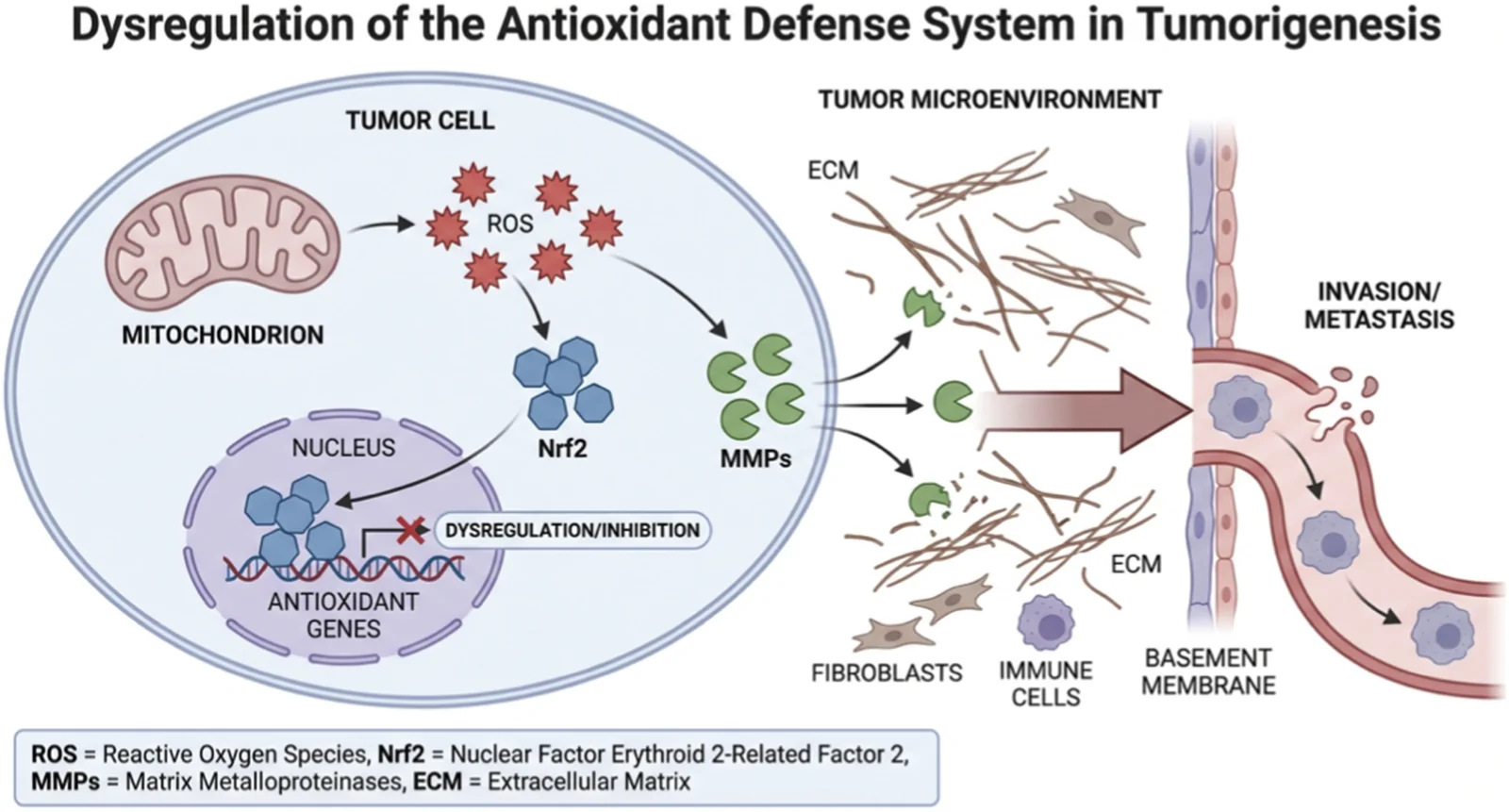
Dysregulation of the antioxidant defense system in tumorigenesis. Mitochondria-derived reactive oxygen species (ROS) influence tumor microenvironment and invasion through Nrf2 and ma-trix metalloproteinases (MMPs). Nox1-mediated increase of ROS affects tumor invasion. ECM, extra-cellular matrix.

Cells have evolved a sophisticated antioxidant system to maintain redox homeostasis. En-zymatic antioxidants such as superoxide dismutase (SOD), catalase (CAT), and glutathione pe-roxidases (GPx) convert ROS into less reactive molecules, limiting oxidative damage. Non-enzymatic antioxidants-including glutathione (GSH) and vitamins C and E-directly scavenge radicals and help regenerate other antioxidants. These defenses are supported by redox buffering and recycling pathways (e.g., NADPH-dependent systems) that restore antioxidant capacity after neu-tralization reactions. When antioxidant defenses are overwhelmed or impaired, oxidative stress can disrupt signaling, damage DNA and proteins, and contribute to inflammation and disease progression (Figure 2).

Nrf2 (NFE2L2) is a master transcription factor that maintains cellular redox homeostasis by inducing antioxidant and detoxification programs (e.g., glutathione synthesis and recycling, NADPH regeneration, and ROS-scavenging enzymes) when oxidative or electrophilic stress disrupts the KEAP1–CUL3 “brake” on Nrf2 (He et al., 2020; Tonelli et al., 2018; Wang L. et al., 2022; Yu and Xiao, 2021; Adinolfi et al., 2023). In cancer, this adaptive circuitry can become persistently activated-often through KEAP1 or NFE2L2 alterations or KEAP1-interacting mechanisms-allowing tumor cells to buffer high intrinsic ROS, support metabolic reprogramming, and sustain survival under hypoxia and oncogenic stress (Lin et al., 2023). As a consequence, hyperactive Nrf2 signaling is frequently linked to more aggressive phenotypes, including enhanced growth, stemness, invasion/metastasis, and resistance to chemotherapy/radiotherapy (partly by maintaining redox balance and limiting lipid peroxidation/ferroptosis). Nrf2 also reshapes the tumor immune microenvironment and has been associated with poorer responses to immune checkpoint blockade in multiple tumor types, high-lighting its broader role in tumor progression beyond redox control (Panda et al., 2025). Mechanistically, under basal conditions, KEAP1 acts as a critical negative regulator of NRF2 by facilitating its ubiquitination and proteasomal degradation. Oxidation of specific cysteine sensors, particularly Cys151, Cys273, and Cys288, within KEAP1 by ROS induces conformational changes, disrupting the KEAP1-NRF2 interaction and allowing NRF2 to translocate to the nucleus, where it heterodimerizes with small Maf proteins and binds to antioxidant response elements (AREs) to initiate the transcription of cytoprotective genes (Castelli et al., 2021). This redox-sensitive cysteine network enables KEAP1 to function as a finely tuned sensor of cellular oxidative status. Consistent with the clinical relevance of this pathway, a 2024 retrospective cohort study showed that KEAP1/NRF2 variants display cancer-type-specific distributions and prognostic effects, with NRF2 ETGE-motif variants associated with poorer outcomes (Iwasaki et al., 2024).

Beyond the Nrf2 axis, multiple antioxidant enzyme systems are frequently co-opted by tumors to buffer reactive oxygen species (ROS) while preserving redox signaling that supports proliferation, dissemination, and treatment tolerance. Superoxide dismutases (SODs), particularly SOD1, convert superoxide to H_2_O_2_ and can facilitate tumor-cell adaptation to microenvironmental stress (e.g., hypoxia/starvation), thereby sustaining survival programs and potentially limiting the efficacy of stress-inducing therapies (König et al., 2024). Downstream H_2_O_2_-removal enzymes such as catalase (CAT) act as key “gatekeepers” of peroxide tone; altered CAT expression/activity has been implicated in shaping oncogenic signaling, invasion-related redox cues, and therapeutic responses, making it a recurrently discussed redox vulnerability (Glorieux and Calderon, 2024). The glutathione (GSH) network (GSH synthesis/regeneration, glutathione peroxidases, and glutathione S-transferases) contributes to both ROS detoxification and xenobiotic/drug handling, and is therefore tightly linked to chemoresistance and broader therapy escape; notably, GSTP1 has been shown to support stemness-associated traits, metastatic capacity, and resistance to targeted tyrosine kinase inhibitors in lung adenocarcinoma models. In parallel, ferroptosis-controlling antioxidants—most prominently GPX4 and GSH-dependent lipid peroxide detoxification-enable tumors to evade oxidative cell death and can underlie resistance to therapies that elevate lipid peroxidation pressure (Zhou Q. et al., 2024). Finally, the thioredod peroxiredoxin (PRDX) systems maintain protein-thiol and peroxide homeo-stasis, and recent work highlights their association with malignant behavior, drug response, and emerging links to antitumor immunity, reinforcing the idea that “backup” antioxidant circuits can become rate-limiting dependencies during progression and treatment.

In summary, the relationship between the dysregulation of the antioxidant defense system and tumorigenesis is multifaceted and involves the production, clearance, and signaling pathways of ROS and interactions with the tumor microenvironment. A deeper understanding of these mecha-nisms can aid the development of new strategies for cancer treatment.

### 3.3 Oxidative stress and tumor cell death

**FIGURE 3:**
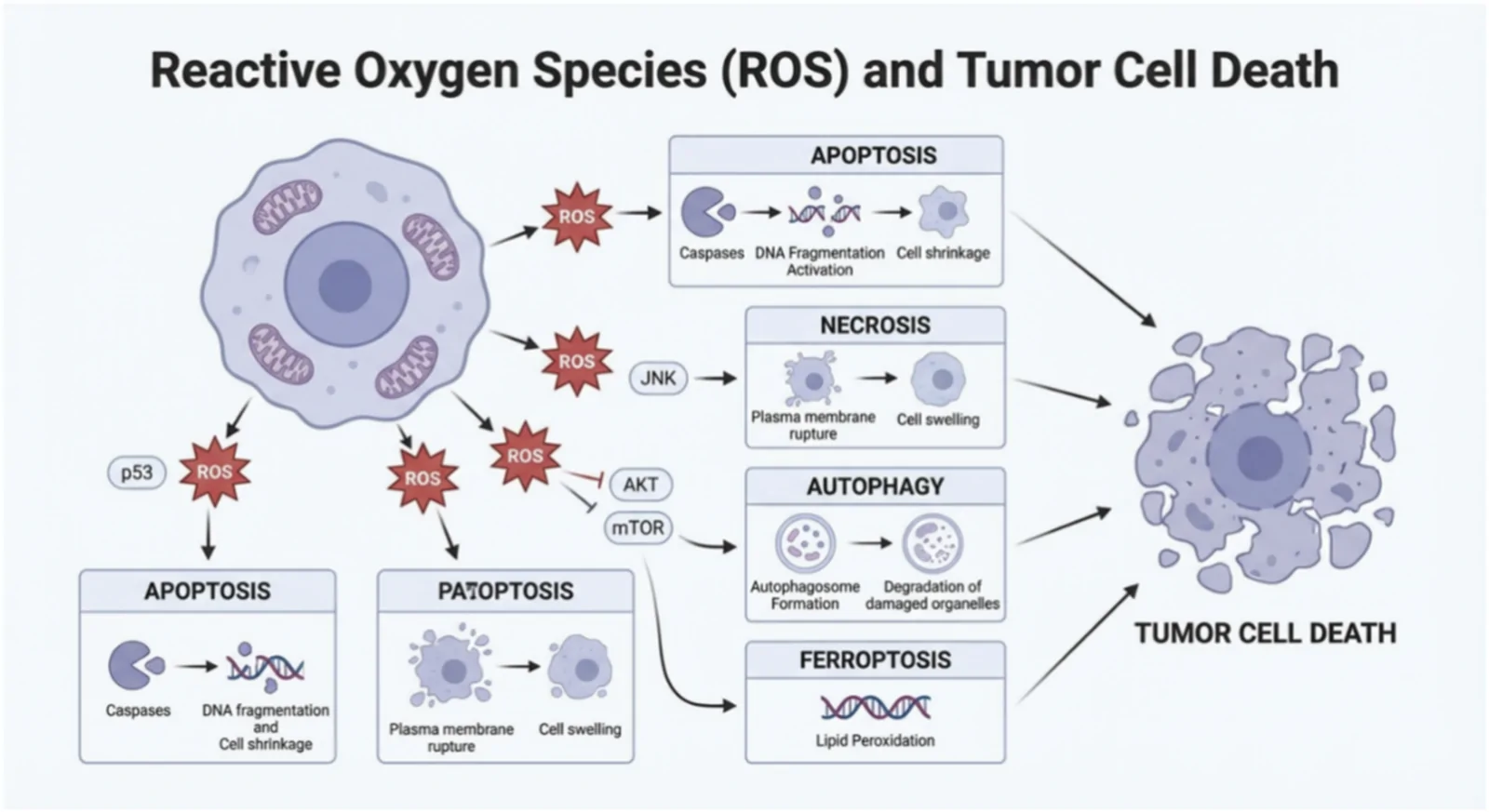
Reactive oxygen species (ROS) and tumor cell death. ROS affects the death of tumor cells through different signaling pathways.

Research indicates that through exogenous pathways, the accumulation of ROS can stimulate cells to increase the expression of antioxidant enzymes, such as SOD, GPX, and catalase, to reduce the destructive effects of ROS, protecting cells from apoptosis. Moreover, through endogenous pathways, the presence of ROS prompts cells to upregulate the expression of antioxidant enzymes, which can effectively clear ROS, alleviate oxidative stress damage, protect the mitochondrial membrane from destruction, prevent the release of cytochrome C, and inhibit the activation of apoptotic signals (Figure 3) (NavaneethaKrishnan et al., 2019; Hu et al., 2021; Imelda et al., 2022; Kang et al., 2023).

ROS can activate the NF-through exogenous pathways, theturn promotes the expression of various antiapoptotic genes, such as Bcl-2, Bcl-xL, and Survivin. These proteins play crucial roles in maintaining mitochondrial membrane stability by preventing the release of cytochrome C, thereby counteracting the apoptotic signals initiated by ROS (Sivandzade et al., 2019). Furthermore, ROS can modulate the interaction between death receptors and their ligands, either directly or indirectly. This includes the binding of Fas ligand (FasL) and tumor necrosis factor (TNF) to their re-spective receptors, Fas receptor and TNF receptor. These interactions facilitate the recruitment of adaptor proteins, such as Fas-associated death domain and TNF receptor-associated death domain, which are instrumental in transforming procaspases into active caspase complexes. This conversion enhances caspase activation, thereby accelerating the apoptotic process in cancer cells (Burdette et al., 2021; Xue et al., 2023). High levels of ROS can also promote the ubiquitination of the antiapoptotic factor FLICE, causing its inhibitory protein FLIP to be degraded or oxidized through the pro-teasome and reducing its competitive inhibition of caspase-8, thereby increasing caspase ac-tivation and upregulating cell apoptosis (Burdette et al., 2021). Concurrently, high levels of ROS activate tran-scription factors, such as p53, promoting the expression of apoptosis-sensitive genes, such as BAX, NOXA, and PUMA, which encode proteins that can promote cell apoptosis. In addition, the activation of p53 can upregulate the expression of FASL, enhancing the interaction with the death receptor FAS and indirectly promoting apoptosis. Further, ROS can directly attack the mitochondrial membrane, causing lipid peroxidation of the mitochondrial membrane, in-creasing its permeability, and prompting the release of cytochrome C from the mitochondrial permeability transition pore into the cytoplasm, activating the caspase pathway and driving cell apoptosis. Under the influence of ROS, p53 can regulate the mitochondrial membrane potential, triggering apoptosis (Srinivas et al., 2019; Liu and Gu, 2022a; Liu and Gu, 2022b).

Recent investigations have revealed that ferroptosis, which is distinct yet analogous to other forms of cell death, is a form of iron-dependent cell death induced by ROS and is typified by the enzymatic or nonenzymatic lipid peroxidation process. The transcription factor NRF2 is activated under low-to-moderate ROS concentrations, subsequently inducing the activation of GSH and GPX4. This regulatory mechanism maintains ROS levels within a manageable threshold, thereby mitigating oxidative stress, curbing lipid peroxide formation, and conse-quently preventing ferroptosis. Furthermore, cancer cells mainly rely on glycolysis instead of oxidative phosphorylation for energy production, which diminishes their need for polyun-saturated fatty acids, which are vulnerable to lipid peroxidation. This metabolic shift conse-quently reduces the likelihood of lipid peroxidation and provides indirect resistance to the threat posed by ferroptosis. Moreover, the cysteine transporter XCT, which is highly expressed in cancer cells, can also inhibit ROS-induced ferroptosis by promoting the uptake of cystine re-quired for GSH synthesis. However, under high levels of ROS, especially highly reactive hy-droxyl radicals and superoxide, cancer cells exacerbate oxidative stress and accelerate the rate of lipid peroxidation. ROS can also promote the release of iron ions from the storage pool into the cytoplasm, where they generate toxic iron through the Fenton reaction, further promoting fer-roptosis. In contrast, high levels of ROS in cancer cells can activate p53, which in turn inhibits NRF2, promoting ferroptosis. Moreover, it can also inhibit the transporter system Xc−, which is composed of SLC7A11 and SLC3A2, leading to reduced GSH synthesis for enhanced ferrop-tosis (Chen et al., 2021; Stockwell, 2022; Zheng et al., 2022). Recent nanomedicine research further demonstrated that simultaneous RRM1 suppression and SLC7A11-GPX4 inhibition can amplify ROS accumulation, lipid peroxidation, and ferroptosis in colon cancer models (Song et al., 2025).

Furthermore, the Keap1have revealed that ferroptosis, which is distinct yet analogous to other forms of cell death, is a form of iron-dependent cell death induced by ROS and is typified bnucleus, where it associates with sMAF proteins and binds to AREs. This interaction initi-ates the transcription of various genes that modulate intracellular ROS concentrations, conse-quently suppressing cellular autophagy. Furthermore, long-term high ROS levels not only trigger the initial autophagy response but may also lead to severe damage to organelles, ex-ceeding the capacity of the autophagy system. Under this extreme situation, the autophagy mechanism may fail or be shut down, failing to effectively address intracellular damage, thereby promoting the survival of cancer cells. Further, ROS accumulation can activate adenosine monophosphate-activated protein kinase (AMPK), which then binds to the ULK1 complex, promoting the process of autophagy. In addition, the activation of AMPK can suppress the PI3K/AKT/mTOR (mammalian target of rapamycin) signaling pathway. Since mTOR serves as a vital inhibitor of autophagy, its suppression promotes the assembly of the autophagy initiation complex, thereby accelerating the autophagic process. Autophagy supplies essential energy and amino acids by degrading and recycling faulty cellular components, thereby restoring cellular redox homeostasis. Moreover, ROS can directly interact with the key autophagy-regulating protein ATG4, thereby facilitating autophagosome formation (Xie et al., 2023; Filomeni et al., 2015; Gu et al., 2020; Hasan et al., 2022; Kma and Baruah, 2022; Li et al., 2015; Wang et al., 2023).

### 3.4 Oxidative stress and the tumor microenvironment

**FIGURE 4:**
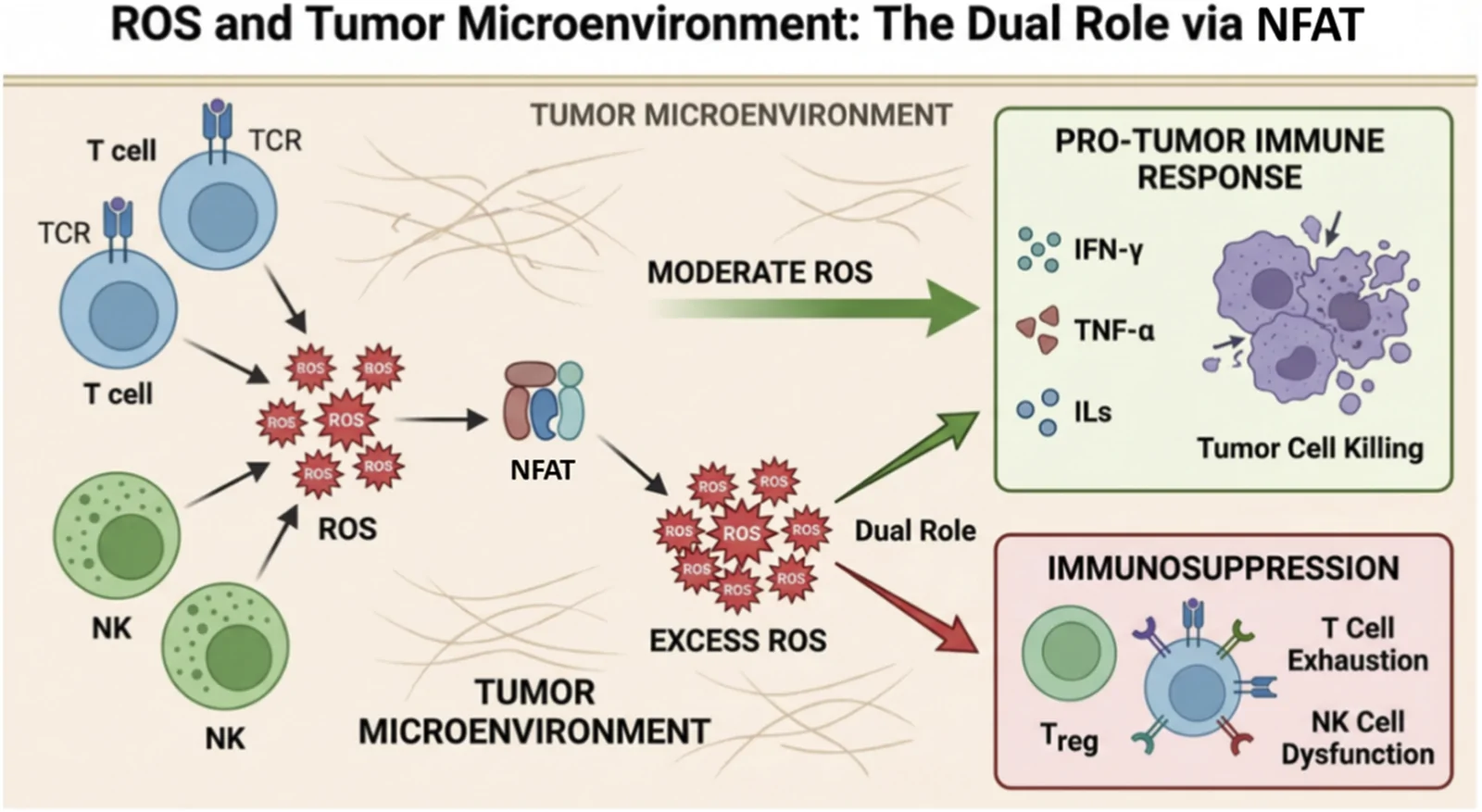
Reactive oxygen species (ROS) and tumor microenvironment. ROS produced by T cells and natural killer (NK) cells promote tumor immune response through NFAT. Excess ROS appears to have a dual role, either promoting immunity or suppressing it. Moderate ROS can mainly promote tumor immune response. IL, interleukin; IFN-γ, interferon gamma; TCR, T-cell receptor; TNF-α, tumor necrosis factor-alpha.

Oxidative stress is a defining biophysical and biochemical feature of the tumor microenvironment (TME), arising from oncogene-driven metabolism, mitochondrial dysfunction, hypoxia–reoxygenation, and ROS production by stromal and immune cells. Persistently elevated ROS and lipid peroxidation products remodel the TME by rewiring redox-sensitive signaling in cancer cells and adjacent stromal compartments, thereby promoting angiogenesis, extracellular-matrix remodeling, and pro-metastatic niche formation (Liang et al., 2025). Importantly, oxidative stress shapes antitumor immunity: ROS can skew myeloid-cell programs toward immunosuppressive phenotypes (including macrophage polarization states) and impair cytotoxic lymphocyte function, collectively weakening immune surveillance and limiting immunotherapy efficacy (Cheng, 2024). At the same time, oxidative stress intersects with regulated cell-death pathways such as ferroptosis, which can be therapeutically leveraged but may also have context-dependent effects on anticancer immunity, underscoring the dual (“Janus-faced”) role of ROS in tumor control versus immune escape (Figure 4).

Activation of T cells occurs at a specific level of ROS. Upon activation, T cells and NK cells produce elevated levels of ROS. Within this framework, ROS act as secondary messengers, playing a key role in the activation of nuclear factor of activated T cells (NFAT) and the suppression of phosphatase activity, thereby maintaining the integrity of signaling pathways. Furthermore, moderate ROS stress can trigger NFAT and increase the secretion of interleukin-2 (IL-2), contributing to the immune response. The generation of ROS following the activation of NK cells can lead to the upregulation of Fas expression while concurrently downregulating the expression of the antiapoptotic gene Bcl-2, ultimately facilitating T-cell apoptosis. Consequently, careful regulation of ROS levels within T cells and NK cells is important to minimize the detrimental effects of excessive ROS. High ROS levels can decrease T-cell receptor (Bekhet and Eid, 2021) abundance and CD16 chain expression, inhibit NF-κB (Cazzola et al., 2021) activation, and consequently impair the production of interferon-γ (IFN-γ), TNF-α, and IL-2 (Chzzola and Tse, 2021; Yang et al., 2023; Yang et al., 2021).

The antioxidant GSH pathway plays an essential role in regulating the redox status of T cells (Muri and Kopf, 2021). A deficiency of GSH in T cells may impair the activation of mTOR and reduce the expression of NFAT and Myc transcription factors, which affects the metabolic reprogramming of T cells during inflammation (Xue et al., 2023; Chowdhury et al., 2020; Esteve and Esteve-Esteve, 2020; Mohan et al., 2023; Pang et al., 2024). During the immune activation process, tumor-specific T cells treated with the antioxidant N-acetyl cysteine can significantly reduce DNA damage and cell death, resulting in better persistence and antitumor efficacy after transfer into mice bearing tumors (Schönrich et al., 2020; Wrotek et al., 2024). Genetically engineered T cells that coexpress chimeric antigen receptors and catalase can not only reduce their own oxidative stress and exert potent antitumor activity but also protect surrounding NK cells from the suppressive effects of ROS. Similarly, NK cells induced by IL-15 can gain antioxidant stress capabilities through the thioredoxin system, which helps protect other lymphocytes within the tumor microenvironment from ROS damage (Srinivas et al., 2019; Cheung and Vousden, 2022).

Further, ROS play a role in the induction of regulatory T cells (Tregs), which can utilize ROS to suppress the activity of other immune cells. In the tumor microenvironment, the number of Tregs often increases, suggesting elevated oxidative stress. Previous studies have shown that Tregs are resistant to oxidative stress, which may be due to their increased antioxidant ability. Further studies have confirmed that GSH deficiency in Tregs leads to alterations in serine metabolism, mTOR activation, and increased cell proliferation, accompanied by downregulation of FoxP3 expression, resulting in reduced suppressive function of Tregs both *in vitro* and *in vivo* (Song et al., 2020; Yang et al., 2023; Dong et al., 2022; Xiao et al., 2021).

Oxidative stress plays a pivotal role in modulating the cytotoxic actions of immune cells. Under physiological conditions, a moderate increase in ROS can upregulate the metabolism, synthesis, and secretion of immune cytokines and various effector molecules (Akbarali et al., 2022; Romani, 2022). In contrast, excessive ROS levels, along with the use of immunosuppressive agents, such as PD-L1 and lactate, can hinder immune cell regulation. Oxidative stress occurring within the endoplasmic reticulum can induce immunogenic cell death (ICD) in cancer cells (Vitale et al., 2013). ICD facilitates the expression and release of damage-associated molecular patterns, including ATP, endoplasmic reticulum-associated calreticulin, and the nuclear protein high mobility group box 1 (Ahmed and Tait, 2020; Birmpilis et al., 2022; Fucikova et al., 2020; Li et al., 2022; Pangilinan et al., 2024). These damage-associated molecular patterns bind to receptors on dendritic cells, such as CD91, TLR4, and P2RX7, leading to the dendritic cell activation, promotion of antigen cross-presentation, and CD8^+^ T-cell response against the tumor (Dong et al., 2022; Bekeschus et al., 2022; Fu et al., 2022; He et al., 2023).

Thus, the impact of ROS on tumor cells and the immune system is largely dependent on their concentration. In immune cells, a physiological increase in ROS levels can enhance metabolism and facilitate the synthesis and secretion of cytokines and other effector molecules. In contrast, elevated ROS levels, along with the use of immunosuppressive agents such as PD-L1 and lactate, exert a suppressive regulatory effect on immune cells. In addition, excess ROS in the tumor microenvironment can impair immune cell function by oxidizing crucial immune molecules and disrupting signal transduction between dendritic cells and T cells (Xue et al., 2023; Murray and Mirzayans, 2021; Bailly, 2020; Glorieux et al., 2021). Mechanistically, genetic or RNA-interference-mediated attenuation of Nrf2 preserved intratumoral CD8^+^ T-cell and CAR-T-cell survival and effector function in ROS-rich tumors and improved tumor control in preclinical solid-tumor models (Jo et al., 2024).

A recent comprehensive review by Shen et al. systematically delineated how oxidative stress remodels the TME by rewiring redox-sensitive signaling in cancer cells and adjacent stromal compartments, thereby promoting angiogenesis, extracellular matrix remodeling, and pro-metastatic niche formation (Shen et al., 2025). Concurrently, Li et al. proposed the “ROS-Immune Mismatch” framework, highlighting that the differential redox adaptation between tumor cells and immune cells, whereby effector T cells are exhausted while immunosuppressive regulatory T cells (Tregs) and M2-like tumor-associated macrophages (TAMs) thrive under oxidative stress, is an active driver of immune evasion (Li et al., 2026). This conceptual advance provides a mechanistic rationale for combining ROS-modulating strategies with immunotherapies. Importantly, this mismatch is not merely a passive consequence of the oxidative TME but an actively selected trait that favors tumor survival and immune evasion (Gilchrist et al., 2026).

### 3.5 ROS-mediated epigenetic regulation

Beyond genetic alterations, ROS have emerged as critical modulators of the cancer epigenome. As recently reviewed by Yang et al., ROS function as key signaling molecules that drive not only tumorigenesis and metabolic reprogramming but also epigenetic remodeling through a bidirectional crosstalk mechanism (Yang et al., 2026). This “metabolism-signaling-epigenetic” feedback loop enables tumor cells to maintain homeostasis and drive adaptive evolution under stress. Mechanistically, ROS influence DNA methylation by modulating the activity of DNA methyltransferases (DNMTs) and ten-eleven translocation (TET) family dioxygenases. ROS can alter the site-specific occupancy of DNMTs and suppress TET-mediated oxidation of 5-methylcytosine (5 mC) by modulating the availability of Fe^2+^ and α-ketoglutarate, cofactors essential for TET activity (Yang et al., 2026). Additionally, ROS can oxidatively modify reader proteins such as MBD family members, compromising the fidelity of methylation maintenance. For example, oxidation of Cys359 in MBD2 disrupts its phase-separation capacity, impairing its function in recruiting the NuRD complex to silence gene expression (Yang et al., 2026). In turn, epigenetic modifications feed back to regulate ROS homeostasis through the control of antioxidant and metabolic pathway genes, establishing a self-reinforcing regulatory circuit (Yang et al., 2026; Tao et al., 2024). Histone modifications are also redox-sensitive. ROS can affect histone-modifying enzymes, including histone deacetylases (HDACs) and demethylases. Oxidation of specific cysteine residues on HDACs can alter their activity, affecting chromatin compaction and gene expression (Tao et al., 2024). Furthermore, ROS stress is known to alter the expression of numerous non-coding RNAs, which can serve as epigenetic regulators. The therapeutic implications of this ROS-epigenetic crosstalk are profound. Combining DNMT inhibitors with modulators of the antioxidant network, or enhancing TET activity to correct aberrant methylation patterns, represents a promising avenue for cancer therapy (Yang et al., 2026). In support of a direct redox-chromatin mechanism, a 2024 study identified H3.1 Cys96 as a chromatin-embedded redox sensor; mitochondrial H_2_O_2_-driven oxidation promoted H3.1 eviction, H3.3 replacement, EMT-associated chromatin opening, metastasis, and multidrug resistance (Palma et al., 2024b).

## 4 Oxidative stress and cancer therapy

The concentration-dependent effects of ROS are increasingly attracting attention in cancer treatment, given the potential to promote the formation of tumors and inhibit their proliferation. ROS can cause DNA damage and genetic mutation, thereby driving the tumor process. In contrast, raising the concentration of ROS within tumor cells can trigger the apoptosis of these cells, achieving the goal of treating cancer (Li et al., 2023; Bell et al., 2024; Martin-Perez et al., 2022). Redox modulators are agents that intentionally alter cellular redox homeostasis. They can act by increasing ROS production, weakening endogenous antioxidant systems such as the GSH-GPX4 and thioredoxin pathways, or, in selected contexts, strengthening antioxidant defenses to limit excessive oxidative injury. In cancer treatment, pro-oxidant agents and antioxidant-system inhibitors are generally intended to push tumor cells beyond their oxidative tolerance threshold, whereas antioxidant approaches may be used more selectively to protect normal tissues or modulate the tumor microenvironment. Redox modulators may also be combined with conventional chemotherapy and radiotherapy, as well as photodynamic therapy and immunotherapy, because these treatments frequently depend, at least in part, on ROS generation or redox-sensitive immune responses. Representative agents, mechanisms, cancer types, and clinical evaluation are summarized in Table 1. A recent systematic review by Sahu and Jain has provided a pathway-oriented classification of ROS-modulating compounds, categorizing them according to their primary actions on ROS-regulated signaling cascades including p53, MAPK/ERK, JNK/p38, PI3K/AKT, Nrf2, NF-κB, DR5/caspase, NLRP3, AMPK, and HIF-1α pathways (Sahu and Jain, 2026). While their classification offers a valuable framework for understanding the mechanistic diversity of ROS modulators, our review extends this analysis by emphasizing the translational status of these agents including their clinical trial phases, combination strategies, and the specific barriers to clinical implementation, thereby bridging the gap between pathway-level understanding and patient-oriented applications.

**TABLE 1:** Selected ROS-targeted therapies in clinical development.

Agent	Class	Target/Mechanism	Cancer type	Phase	NCT identifier
APX3330	APE1/Ref-1 inhibitor	Blocks redox signaling; inhibits NF-κB and HIF-1α	Advanced solid tumors	Phase I	NCT03375086
Setanaxib (GKT137831)	NOX1/4 inhibitor	Inhibits NOX4 in CAFs; restores T-cell infiltration	Head and neck squamous cell carcinoma	Phase II	NCT05323656
Elesclomol	Copper ionophore	Induces mitochondrial ROS; cuproptosis	Melanoma	Phase III	NCT00522834
High-dose vitamin C	Pro-oxidant	Generates H_2_O_2_ via iron-dependent fenton reaction	Pancreatic cancer	Phase II	NCT02905578
Buthionine sulfoximine (BSO)	GSH synthesis inhibitor	Inhibits γ-glutamylcysteine synthetase; depletes GSH	Neuroblastoma	Phase I	NCT00005835
Auranofin	TrxR inhibitor	Inhibits thioredoxin reductase; promotes ROS accumulation	Lung cancer, ovarian cancer	Phase II	NCT01737502

### 4.1 Oxidative stress as a target in cancer therapy

The effects of ROS on cancer metabolism, immune responses, phase separation, cancer stem cells, and cell fate have emerged as prominent research topics. Significant progress has been made in the development of therapeutic strategies that target ROS. Evidence suggests that ROS can activate redox-sensitive signaling pathways in tumor-associated macrophages, prompting the release of inflammatory mediators that influence tumor progression and metastasis. In addition, IFN-γ secreted by T cells has been shown to downregulate the expression of SLC7A11 and SLC3A2, crucial proteins involved in promoting cysteine consumption via cystine transport mechanisms (Frisoli et al., 2020). Compared with M2 macrophages, M1 macrophages are more resistant to drugs that induce ferroptosis, and inhibiting inducible nitric oxide synthase may be a new strategy for regulating macrophage ferroptosis. Nevertheless, the therapeutic application potential of this regulatory mechanism requires further research and exploration (Cai et al., 2023; Yu et al., 2023).

The induction of oxidative stress is under active research as a strategy to suppress tumor cell proliferation and induce cell death. This can be achieved by either increasing ROS production or impairing cellular antioxidant defense mechanisms. Prior research has demonstrated that the activation of the RAS signaling pathway can upregulate NADPH oxidase (NOX) family enzymes, leading to elevated ROS levels-a phenomenon observed across multiple oncogenic processes. Given the high expression of CD44 in various cancer cells, NOX inhibitors that target CD44, such as GKT831 encapsulated in hyaluronic acid nanoparticles, have shown significant anticancer effects in various animal models. Considering the high expression of CD44 and NOX4 in cancer stem cells, future research should focus on evaluating the effects of these NOX inhibitor nanocarriers on cancer stem cells (Liu J. et al., 2022; Pecchillo Cimmino et al., 2023; Vermot et al., 2021).

Recently, several research teams have successfully developed an innovative photooxidation therapy that utilizes a special photosensitizer to effectively kill cancer cells under the excitation of near-infrared light. This therapy induces cancer cell death through a unique mechanism, effectively overcoming the issue of drug resistance in cancer cells. VVD-130037 (BAY 3605349), a KEAP1 activator developed by Vividion Therapeutics, a subsidiary of Bayer, has entered phase I clinical trials to evaluate its safety, pharmacokinetic properties, pharmacodynamic characteristics, and preliminary efficacy in patients with advanced solid tumors (Kato et al., 2021; Liu et al., 2021; Wakiyama et al., 2021). Moreover, studies on organosilicon-based hollow bilirubin nanoparticles have introduced a novel nanoantioxidant capable of carrying both glucose oxidase and triazine photosensitizers. Through an antioxidant-activated self-protection mechanism and a tumor-specific hypoxic environment-driven synergistic therapeutic strategy, the nanoantioxidant significantly inhibits the growth of solid tumors. These research cases highlight the great potential and broad application prospects of oxidative activators in the field of cancer treatment (Khalil et al., 2019). More recently, nitric oxide-releasing zinc phthalocyanines were shown to reduce mitochondrial oxygen consumption, enhance photodynamic therapy under hypoxic conditions, and induce immunogenic cell death and antitumor immune activation in preclinical models (Xu F. et al., 2024).

Furthermore, the prospects and challenges of ROS-based antitumor therapy include the conflicting preclinical and clinical results of redox modulation therapy strategies, which require careful evaluation of the similarities and differences between different experimental models and human redox regulation. The unregulated induction of oxidative stress by drugs, which can lead to nonspecific damage to healthy tissues, along with the resistance exhibited by certain cancer cells, poses significant challenges to the broader implementation of these therapies (Guan et al., 2024; Nakamura and Takada, 2021).

**FIGURE 5:**
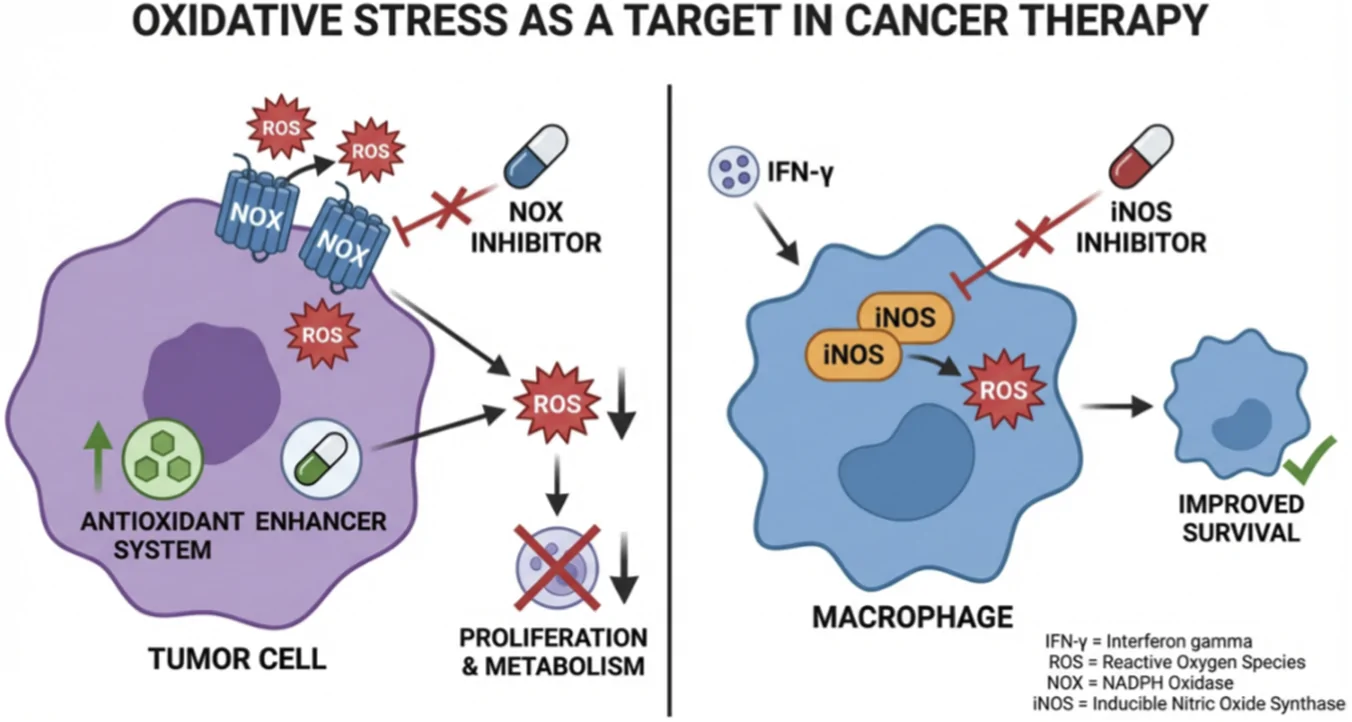
Oxidative stress as a target in cancer therapy. By inhibiting NADPH oxidase (NOX) family enzymes and enhancing the antioxidant system, tumor cell proliferation and metabolism are inhibited. Inducible nitric oxide synthase (iNOS) inhibition is beneficial to the survival of macrophages. IFN-γ, interferon gamma; ROS, reactive oxygen species.

In summary, ROS-directed therapeutics offer promising avenues for oncological intervention. However, forthcoming research is imperative to elucidate the multifaceted roles of ROS throughout various stages of tumorigenesis. In addition, the targeting of ROS-mediated signaling cascades should be refined to improve therapeutic outcomes and mitigate adverse effects (Figure 5).

### 4.2 The controversy surrounding the use of antioxidants in cancer therapy

The role of antioxidants in cancer treatment has been controversial, largely because of the concentration-dependent effects of ROS. On one hand, ROS can promote tumor development by oxidizing specific chemical groups in cells, causing genetic mutations and activating related biochemical pathways (Hardy et al., 2018; Levinn et al., 2019; Sharma et al., 2022), thereby promoting cell proliferation and oncogenic transformation. In contrast, antioxidants may inhibit the proliferation and metastasis of cancer cells by scavenging ROS (Apostolova et al., 2020; Gyanwali et al., 2022; Yi et al., 2023). Antioxidants may disrupt the immune system’s surveillance and clearance of cancer cells by neutralizing physiological levels of ROS, thereby promoting tumor development. The use of antioxidants should be individually assessed and monitored according to the cancer stage, redox status of cancer cells, immune function of individual patients, and corresponding interventions (Kawagishi and Finkel, 2014). The balance between ROS and antioxidants plays a significant role in the plasticity of cancer stem cells (CSCs). Low levels of ROS maintain the resting state of CSCs, whereas high levels of ROS promote the transition of stem cells to the proliferative state. These findings indicate that the levels of ROS and the use of antioxidants need to be finely regulated to avoid activating CSCs and advancing tumor progression (Ngo et al., 2019; Xiao et al., 2018).

The potential side effects of using oxidants to treat cancers are also highly controversial. Oxidation activators attack cancer cells by increasing ROS levels, but this process can also cause oxidative damage to normal cells, especially those that are metabolically active. Excessive ROS can lead to immune cell dysfunction, hindering signaling between dendritic cells and T cells and weakening the ability of the immune system to attack tumors. In addition, the use of oxidation activators may lead to drug resistance in cancer cells, especially when they activate antioxidant pathways, such as NRF2, which help cancer cells to survive and develop resistance under oxidative stress. Under certain conditions, ROS may promote the metastasis of cancer cells, particularly in the late stages of cancer. Further, studies have shown that supplementation with antioxidants may increase the risk of autoimmune diseases, such as multiple sclerosis, lupus erythematosus, and rheumatoid arthritis. In mouse models of non-small cell lung cancer, specific antioxidants, including N-acetylcysteine and vitamin E, have been shown to potentially increase cancer burden and mortality rates in a dose-dependent manner. Oxidative activators may also influence the effects of various cancer treatments, as part of the mechanism of radiotherapy involves killing cancer cells through oxidative damage. Given the potential side effects of oxidative activators in cancer treatment, personalized assessments should be made according to the specific conditions of the patients, with the patients’ reactions and side effects closely monitored to ensure the safety and effectiveness of the treatment (Racanelli et al., 2018; Hou et al., 2020; Nabavi-Rad et al., 2022).

The SELECT randomized prevention trial showed that high-dose vitamin E supplementation increased prostate cancer risk, whereas observational data from the MARIE cohort indicated that antioxidant supplement use during chemotherapy or radiotherapy was associated with poorer outcomes in postmenopausal women with breast cancer (Akter et al., 2026). These findings underscore that systemic antioxidant administration is not universally beneficial and may, in some contexts, promote tumor progression by shielding cancer cells from ROS-mediated cytotoxicity (Gilchrist et al., 2026). The controversy surrounding the use of antioxidants in tumor treatment mainly encompasses their potential pro-tumor effects and how to precisely regulate ROS levels to optimize their therapeutic effects. Future studies should comprehensively consider the effects of ROS on both cancer cells and the immune system and tailor antioxidant treatment strategies based on the distinct characteristics of the tumor and individual patient differences. Conversely, a randomized phase II trial in metastatic pancreatic cancer found that pharmacological ascorbate combined with gemcitabine and nab-paclitaxel prolonged survival, emphasizing that dose, route, tumor context, and combination regimen determine whether vitamin C acts predominantly as an antioxidant or a pro-oxidant (Bodeker et al., 2024).

### 4.3 Application of oxidative stress inducers in cancer treatment

In addition to endogenous sources, cancer cells are exposed to exogenous ROS from environmental and therapeutic sources. Key exogenous sources include: (1) ionizing radiation, which induces ROS production via water radiolysis; (2) chemotherapeutic agents (e.g., cisplatin, doxorubicin), which generate ROS through mitochondrial dysfunction and NADPH oxidase activation; (3) environmental pollutants, such as particulate matter (PM2.5) and heavy metals, that activate inflammatory pathways and ROS production; and (4) specific dietary components and phytochemicals that can act as pro-oxidants (Sahoo et al., 2022; Akter et al., 2026). Shikonin, for example, was reported in 2024 to suppress anaplastic thyroid carcinoma by promoting ROS-associated ferroptosis and inhibiting PKM2-dependent glycolysis (Yang C. et al., 2024).

The relatively high levels of oxidants in cancer cells provide a theoretical basis for dual therapeutic strategies that modulate redox status (pro-oxidant therapy and/or antioxidant therapy). Compared with normal cells, cancer cells are more sensitive to ROS. Targeting the redox vulnerability of cancer cells via the use of pro-oxidants that can produce excessive ROS has become an important anticancer strategy. This approach includes stimulating the production of ROS and/or inhibiting the antioxidant system to induce oxidative stress, leading to cellular damage, inhibition of cell proliferation, and cell death. Recent research has discovered new redox signaling pathways and novel anticancer agents that target ROS. The impact and application of these research advances in tumor treatment and drug development have been extensively discussed, and new concepts, controversial hotspots, and future research directions in the field of ROS have been analyzed (Moloney and Cotter, 2018; Aiello et al., 2019).

Although redox regulation is considered a promising strategy for cancer treatment, the preclinical and clinical outcomes are contradictory, necessitating careful assessment of the similarities and differences in experimental models and human redox regulation. Several challenges have been encountered, including why cancer cells and normal cells respond differently to the same ROS regulators, the controversial role of antioxidants in cancer treatment, and the limitations of analytical methods in detecting redox changes *in vivo*.

### 4.4 Synergistic effects of oxidative stress and chemotherapeutic drugs

The synergistic effect of oxidative stress and chemotherapy is also a hot topic in cancer treatment. The cytotoxicity of chemotherapeutic drugs and the antitumor immune response can be enhanced by targeting the disruption of redox homeostasis in cancer cells. For example, the newly designed and synthesized compound Cu-1 can selectively exert strong cytotoxic effects on cancer cells by targeting the redox balance and stimulating the chemotherapeutic immune response of the cancer cells. In addition, chemotherapeutic drugs, such as arsenic trioxide and gemcitabine, can induce tumor cell apoptosis by increasing the generation of intracellular ROS, increasing Bax gene expression, and inhibiting the activity of the nuclear transcription factor-κB. However, cancer cells can develop resistance to chemotherapeutic drugs by reducing the production of ROS. Photodynamic therapy can increase intracellular ROS production, leading to the death of chemoresistant cells and thus inhibiting the chemo-resistance of tumor cells (Hossain et al., 2022; Peng et al., 2017; Calvani et al., 2019; Chen et al., 2020; Sahoo et al., 2022). Likewise, NUAK1 inhibition was shown to induce ROS-dependent immunogenic cell death, whereas co-targeting the mevalonate pathway with simvastatin intensified this response and improved anti-PD-1 activity in tumor models (Gui et al., 2025).

### 4.5 Oxidative stress and cancer treatment resistance

Drug resistance is a significant and longstanding challenge in cancer treatment. Oxidative stress plays a complex role in chemotherapy resistance in cancer through various mechanisms. Tumor cells may suppress ferroptosis by increasing their ability to resist oxidative stress, leading to the development of resistance. In clear cell renal cell carcinoma, DPP9 can inhibit ferroptosis by stabilizing NRF2 protein levels and activating oxidative stress signals, thus increasing resistance to targeted therapy. In addition, cancer immunotherapy can alter the redox state of tumors, aggravate oxidative stress, and trigger ROS-dependent cancer rejection reactions (Slade, 2020; Niu et al., 2021).

The role of ROS in drug resistance is intimately connected to their pro-metastatic functions. As reviewed by Gilchrist et al., ROS promote metastasis through multiple interconnected mechanisms: they drive epithelial-mesenchymal transition via NOX activation and HIF-1α stabilization, enhance tumor cell migration by inactivating redox-sensitive phosphatases (e.g., PTEN, PTP1B), promote invasion through MMP2/MMP9 upregulation, and facilitate circulating tumor cell survival via NOX4-mediated anoikis resistance (Gilchrist et al., 2026). Importantly, the same ROS-dependent survival mechanisms that enable CTCs to withstand detachment-induced apoptosis also contribute to chemotherapy resistance in primary tumors, suggesting that targeting ROS-dependent survival pathways may simultaneously inhibit metastasis and overcome drug resistance.

Researchers have recently developed nanomedicines designed to promote ROS homeostasis by increasing fatty acid oxidation and suppressing autophagy. These advancements have the potential to overcome cancer drug resistance and promote tumor cell apoptosis, as demonstrated in both *in vitro* and *in vivo* experiments. In addition, studies indicate that hydrogen sulfide can increase the sensitivity of non-small cell lung cancer cells to ferroptosis by lowering homocysteine levels and inhibiting the transsulfuration pathway, suggesting a promising new strategy for lung cancer treatment (Oumeddour et al., 2024; Khattak et al., 2022; Murphy et al., 2019).

In addition to the aforementioned nanomedicines and hydrogen sulfide, other studies are exploring how to harness oxidative stress to overcome drug resistance in cancer treatment. For example, some research teams are investigating specific compounds designed to preferentially activate oxidative stress responses in cancer cells by exploiting tumor-associated metabolic and signaling dependencies. These compounds typically achieve their selective effects by targeting metabolic pathways or signaling pathways unique to cancer cells. Furthermore, advancements in gene-editing techniques, particularly the CRISPR/Cas9 system, have enabled researchers to precisely target and modify genes involved in antioxidant defense mechanisms. Knocking out or downregulating specific genes, such as GPX4, can significantly increase cancer cell susceptibility to oxidative stress. For example, deletion of the GPX4 gene markedly enhanced the vulnerability of cancer cells to ferroptosis, suggesting new therapeutic possibilities (Chen et al., 2019; Hryhorowicz et al., 2017; Janik et al., 2020; Wang S. W. et al., 2022; Forcina and Dixon, 2019; Liu Y. et al., 2023; Ma et al., 2022; Seibt et al., 2019; Ursini and Maiorino, 2020). Recent studies have provided more specific evidence supporting this strategy. The small-molecule degrader ZX703 reduced GPX4 abundance through both the ubiquitin-proteasome and autophagy-lysosome pathways, leading to lipid ROS accumulation and ferroptosis in cancer cells. In ovarian cancer models, combined inhibition of NRF2 and GPX4 increased ROS and lipid-peroxidation products and synergistically suppressed tumor spheroid formation and intraperitoneal tumor growth. In addition, auranofin was shown to convert thioredoxin reductase from an antioxidant enzyme into a ROS-generating protein while simultaneously disrupting mitochondrial respiration and glycolysis, resulting in energy depletion and antitumor activity in lymphoma models. These findings provide concrete examples of how tumor-associated antioxidant and metabolic dependencies may be exploited to enhance oxidative stress and overcome treatment resistance. Nevertheless, these strategies remain predominantly preclinical, and their tumor selectivity, normal-tissue toxicity, pharmacokinetics, and predictive biomarkers require further investigation (Hu et al., 2024; Li N. et al., 2024; Yang M. et al., 2024).

These studies reveal a complex interaction between oxidative stress and drug resistance in cancer therapy, indicating that modulating oxidative stress responses may provide new strategies for cancer treatment. However, these studies are still in their preliminary stages, and further clinical research is needed to verify their potential and effectiveness in cancer treatment.

## 5 Challenges and prospects of oxidative stress in cancer treatment

Therapeutic strategies aimed at modulating ROS present significant potential for advancing cancer treatment, introducing both new opportunities and challenges. Recent research highlights the intricate interplay between redox signaling and cancer metabolism in cancer cells, elucidating the roles of ROS in regulating epigenetic changes and influencing the transcription of messenger RNAs and long noncoding RNAs. In addition, the effects of redox processes within the tumor microenvironment and immune cells provide critical insights. These findings not only enhance our understanding of the fundamental biology of redox mechanisms but also offer valuable perspectives on the aberrant biochemical processes in malignant cells and their complex interactions with host biological systems (Fu et al., 2018; Huang et al., 2023; Zhang Y. et al., 2024).

Despite extensive preclinical evidence, the clinical translation of ROS-targeted therapies faces major hurdles. First, patient stratification remains a critical challenge: the lack of validated biomarkers to assess a patient’s baseline redox status or predict response to therapy limits the ability to select appropriate candidates. Second, off-target toxicity is inherent to ROS modulation, as the induction of oxidative stress is non-specific and can damage normal tissues, particularly the heart, liver, and kidneys (Akter et al., 2026). Third, NRF2-driven resistance is a major obstacle: many tumors harbor constitutively active NRF2 signaling (e.g., via KEAP1 loss-of-function mutations), making them highly resistant to pro-oxidant therapies (Xu et al., 2025). Fourth, the spatiotemporal complexity of ROS regulation such as different ROS species, distinct subcellular sources, and varying kinetics of production makes it difficult to achieve precise therapeutic modulation (Akter et al., 2026).

Experiments across various models have demonstrated dose-dependent effects of ROS on cancer cells, revealing that ROS can exert differing, and sometimes even opposing, outcomes depending on their concentration. This insight offers a plausible explanation for the longstanding paradox regarding the effects of ROS on cancer cells, partially resolving this complex issue. Despite the clarity of the three-tier concentration-dependent model *in vitro*, translating these findings into clinical practice faces significant challenges. Firstly, intratumoral heterogeneity leads to a mosaic of ROS levels, making it difficult to target a specific “dose” of ROS. Secondly, the lack of non-invasive, real-time ROS sensors for *in vivo* use limits our ability to monitor and stratify patients. Finally, *in vitro* models often lack the complexity of the TME, particularly the contribution of stromal and immune cells to the overall redox state (Akter et al., 2026; Gilchrist et al., 2026). Additionally, recent advances in understanding the mechanisms of ROS redox signaling and their concentration-dependent impacts lay a critical foundation for devising more effective therapeutic strategies. These strategies involve the use of ROS-modulating compounds, either alone or in rational combination with other treatments. Continued research in this domain is likely to revolutionize cancer therapy (Heinhuis et al., 2019; Khan et al., 2021; Payandeh et al., 2020; Semlali et al., 2021).

The significant variations in the dynamic changes and regulatory mechanisms of redox status between normal and cancer cells, along with their differing capacities to respond to oxidative stress and ROS modulators, provide a theoretical basis for designing novel, selective anticancer compounds. While numerous studies have consistently demonstrated that cancer cells exhibit greater sensitivity to ROS generators than normal cells do, the precise molecular and biochemical mechanisms driving this disparity remain elusive. Recent research has revealed distinct differences in the IncRNA transcription profiles between normal and cancer cells exposed to ROS stress, which may partly account for the observed differences in sensitivity (Ferrer and Garcia, 2022; Gao et al., 2023; Kozłowska-Masłoń et al., 2022). These lncRNAs exert their functions through various mechanisms, including acting as competing endogenous RNAs (ceRNAs) to sponge miRNAs, serving as scaffolds for protein complexes, and modulating chromatin states (Yang et al., 2026). However, the study of lncRNAs in redox biology is still in its infancy. Limitations include the lack of functional validation, the challenge of distinguishing causality from correlation, and their generally low conservation across species. These differences may partly account for the observed differences in sensitivity between normal and cancer cells to ROS stress.

Research breakthroughs in ROS-targeted cancer therapies rely on the use of suitable research models, availability of precise techniques for ROS analysis, and effective application of tools designed to detect and quantify diverse types of ROS within biological systems. A significant obstacle persists, as some researchers struggle to distinguish among various ROS types, often leading to the inappropriate use of “ROS dyes” or probes that fail to identify the specific ROS under examination. While this technical nuance might appear insignificant, it can result in considerable misconceptions and culminate in flawed research outcomes. Nevertheless, recent studies provide crucial guidance for the accurate measurement of ROS and evaluation of oxidative damage in biological systems, thereby improving the robustness of ROS detection and interpretation of the resulting data.

Ongoing advancements in analytical technologies, particularly those capable of measuring ROS within subcellular organelles and even at a specific level *in vivo*, are poised to spur more research on ROS metabolism and signaling pathways. Moreover, the integration of “omics” technologies with redox analysis to characterize alterations in RNA, protein structures, epigenetic modifications, and metabolite profiles under ROS stress has the potential to reveal intricate mechanisms. Such research will deepen our understanding of the role played by ROS in the initiation and progression of cancer, thus laying a robust scientific foundation for the development of novel therapeutic strategies (Oparka et al., 2016). An ROS-responsive manganese-bilirubin nanoparticle magnetic resonance imaging probe has enabled noninvasive mapping of intratumoral ROS and simultaneous monitoring of drug delivery *in vivo*, illustrating the potential of theranostic redox imaging (Jung et al., 2024).

In cancer treatment research, the role of antioxidants remains a topic of considerable debate. Various research groups have reported conflicting findings regarding the influence of antioxidants on tumor growth, with some studies revealing completely opposite outcomes. Recent investigations have illuminated the varying impacts of redox regulation and antioxidants on both immune and cancer cells, offering crucial mechanistic insights into the potential dual nature of the antitumor effects of antioxidants. This bidirectional action could partly clarify the longstanding controversies observed in experimental models across various stages of cancer progression and different states of immune function (Gan, 2021; Sahai et al., 2020; Yang et al., 2022).

Future research endeavors are anticipated to reveal additional molecular targets related to oxidative stress and redox signaling via high-throughput screening and advanced bioinformatics analyses. By leveraging individual variations in redox status, more personalized therapeutic regimens could be formulated. Moreover, investigating the combination of redox modulators with traditional anticancer agents offers a promising strategy to increase therapeutic efficacy and reduce adverse effects. A deeper understanding of the role of oxidative stress in the development of drug resistance is essential for the development of novel strategies to combat this challenge. Bridging the gap between laboratory findings and clinical practice will require comprehensive clinical trials to confirm the safety and effectiveness of these innovative treatments.

## 6 Conclusion

Oxidative stress plays a central yet highly context-dependent role in cancer initiation, progression, and treatment response. Rather than exerting a simple binary effect, ROS function according to a three-tier concentration-dependent model. Basal ROS levels are required for physiological redox signaling and the maintenance of cellular homeostasis. Moderately elevated ROS levels, which are commonly sustained in cancer cells, promote oncogenic signaling, metabolic adaptation, proliferation, invasion, immune evasion, and resistance to therapy. By contrast, excessive ROS accumulation overwhelms antioxidant defenses, causes irreversible damage to DNA, proteins, lipids, and organelles, and ultimately triggers regulated cell death.

This three-tier framework highlights that successful ROS-targeted therapy should not merely increase or decrease ROS indiscriminately. Instead, therapeutic strategies should be designed either to suppress the adaptive, tumor-promoting effects of moderately elevated ROS or to push tumor cells beyond their oxidative tolerance threshold while minimizing injury to normal tissues and immune cells. Achieving this goal will require improved assessment of tumor redox states, identification of predictive biomarkers, real-time monitoring of ROS dynamics, and rational combination strategies that account for intratumoral heterogeneity and NRF2-driven resistance. A more precise understanding of the concentration-, cell type-, and context-dependent effects of ROS may ultimately enable the development of safer and more effective redox-based precision oncology strategies.
